# Bacteriophage Mediates Efficient Gene Transfer in Combination with Conventional Transfection Reagents

**DOI:** 10.3390/v7122951

**Published:** 2015-12-08

**Authors:** Amanda Donnelly, Teerapong Yata, Kaoutar Bentayebi, Keittisak Suwan, Amin Hajitou

**Affiliations:** 1 Phage Therapy Group, Division of Brain Sciences, Department of Medicine, Imperial College London, Hammersmith Hospital Campus, London W12 0NN, UK; donnela1@tcd.ie (A.D.); teerapong@nanotec.or.th (T.Y.); keittisak.suwan@imperial.ac.uk (K.S.); 2 National Nanotechnology Center, National Science and Technology Development Agency, 111 Thailand Science Park, Pathum Thani 12120, Thailand; 3 Laboratory of Genetics, University of Balearic islands, Valldemossa Road Km. 7,5, 07122 Palma, Spain; kaoutar.bentayebi@gmail.com

**Keywords:** Bacteriophage, phage-based gene delivery, phage biotechnology, transfection, calcium phosphate

## Abstract

The development of commercially available transfection reagents for gene transfer applications has revolutionized the field of molecular biology and scientific research. However, the challenge remains in ensuring that they are efficient, safe, reproducible and cost effective. Bacteriophage (phage)-based viral vectors have the potential to be utilized for general gene transfer applications within research and industry. Yet, they require adaptations in order to enable them to efficiently enter cells and overcome mammalian cellular barriers, as they infect bacteria only; furthermore, limited progress has been made at increasing their efficiency. The production of a novel hybrid nanocomplex system consisting of two different nanomaterial systems, phage vectors and conventional transfection reagents, could overcome these limitations. Here we demonstrate that the combination of cationic lipids, cationic polymers or calcium phosphate with M13 bacteriophage-derived vectors, engineered to carry a mammalian transgene cassette, resulted in increased cellular attachment, entry and improved transgene expression in human cells. Moreover, addition of a targeting ligand into the nanocomplex system, through genetic engineering of the phage capsid further increased gene expression and was effective in a stable cell line generation application. Overall, this new hybrid nanocomplex system (i) provides enhanced phage-mediated gene transfer; (ii) is applicable for laboratory transfection processes and (iii) shows promise within industry for large-scale gene transfer applications.

## 1. Introduction

Gene delivery is one of the most fundamental techniques of molecular biology, and a technological basis for *in vitro* and *in vivo* gene therapy. By definition, gene transfection involves the delivery of nucleic acids (DNA or RNA) into cells to genetically modify them. Expression of transgenes in cell cultures creates a suitable system to determine the regulation and function of a desired gene, and in turn the function of proteins and their network systems. Additionally, transfection has revolutionized scientific industries allowing for the development of large-scale recombinant protein production including antibodies, vaccines and viral vectors [1,2].

Methods developed for transfection can broadly be classified into three categories; biological, chemical and physical [3]. The choice of method depends heavily on the type of system to be transfected, the size of the transgene and whether the resulting output requires transient or stable transgene expression. To achieve successful gene transfer, nucleic acids have to overcome several cellular barriers including surface adsorption and entry, degradation during intracellular trafficking and finally be able to induce transgene expression within the nucleus. Traditionally, transfection of cell cultures is achieved by the use of chemical transfection reagents, which deliver the nucleic acids into cells. Calcium phosphate was the first transfection reagent to be developed and works on the basis that positively charged calcium ions bind to the negatively charged phosphate backbone of DNA and form a co-precipitation complex for transportation into cells through endocytosis [4]. Since calcium phosphate, many different transfection reagents have been developed including cationic lipids (most popular), polycationic polymers [5], and cationic amino acids [6]. All these transfection reagents work on the same basic principal in that the positively charged chemicals interact and condense the negatively charged DNA to form positively charged complexes for easy transport through the negative cell membranes. A successful transfection reagent should have minimal cytotoxicity, high transfection efficiency, be easy to reproduce and be inexpensive, particularly for large-scale transfection processes in industry. However, as with every technology there are limitations; transfection reagents have low efficacy, can be expensive and it is difficult to target them to specific cell types.

On the other hand, viral vectors have also been developed for gene delivery purposes within laboratory research but are mainly used *in vivo* for gene therapy applications. The most successful viral vectors to date include adenovirus, lentivirus and adeno-associated virus [7,8,9,10,11]. While these vectors are superior in their gene delivery efficacy compared with non-viral vectors, they have various limitations. Firstly, they can have a limited packing capacity, which restricts the size of the transgene that can be engineered into their genome. Secondly, they have a complex protein structure, which makes their production complicated, less efficient and very expensive. Finally, they are not deemed safe for applications such as production of recombinant proteins for human purposes as they have a broad tropism for mammalian cells.

Bacteriophages (phage), viruses that infect only bacteria, are attracting increasing attention as promising new biomaterials in the field of gene delivery. Mainly, filamentous M13 bacteriophages are being developed as a new type of vectors for safe and targeted systemic administration of transgenes for *in vivo* applications [12,13,14,15]. They have a number of advantages over the use of traditional viral and non-viral vectors; firstly, their protein coat consists of a repeating protein unit arranged in an alpha helical array. This bestows the phage with an unlimited DNA packaging capacity, as the capsid coat merely needs to elongate to accommodate the transgene. Therefore, the bacteriophage is efficient at condensing and packing the DNA. Secondly, the protein coat has a high tolerance for mutations, allowing peptide ligands to be easily introduced to achieve ligand-directed transduction of the desired cell type. Additionally, they are safe as they have long been administered to humans for the treatment of bacterial infections and have been approved by the Food and Drug Administration (FDA-USA) for use in food preparations [16,17]. Lastly, phage vectors are easy to produce at high titers and at low costs, which is highly desirable for large scale industrial processes. Previously, we reported the development of an M13 phage-based vector consisting of a mammalian transgene cassette flanked by inverted terminal repeats (ITR) from adeno-associated virus serotype 2 (AAV2), incorporated in an intergenomic region of the phage genome. Because bacteriophages have no tropism for mammalian cells, and are therefore unable to enter and transduce these cells, we genetically engineered the phage to display the double cyclic RGD4C (CDCRGDCFC) ligand, on the phage capsid, to bind to αv integrin receptors on the cell surface to allow phage internalization and subsequently expression of the gene of interest. This vector, named AAV/phage or AAVP, has shown promise for specific tumor targeting *in vitro* and *in vivo* [14]. However, bacteriophages remain poor delivery vectors in comparison to traditional viral vectors, as they have no intrinsic strategies for mammalian cell transduction. Therefore, strategies need to be explored to allow efficient phage binding, entry into mammalian cells and subsequent increase of their gene transfer ability, which should subsequently develop their use in a wide range of gene transfer applications.

**Figure 1:**
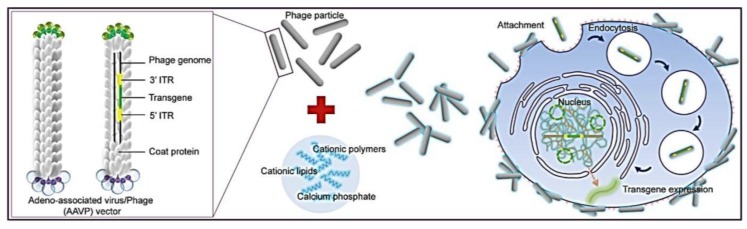
Schematic diagram of the hybrid bacteriophage/transfection reagents system. Negatively charged bacteriophage vectors were electrostatically mixed with transfection reagents to form hybrid bacteriophage complexes. A hybrid vector consists of the filamentous M13 bacteriophage and a transfection reagent. The phage serves as a transgene carrier. ITR, inverted terminal repeats of adeno-associated virus serotype 2 (AAV2).

Combination of viral vectors with traditional transfection reagents has previously been reported for the development of *in vivo* gene delivery vectors [18,19]. Here, we report a new hybrid bacteriophage-based system for general gene delivery processes. We have taken two of the central themes of gene transfer, biological (phage) and chemical (transfection reagents), and combined them into a superior gene delivery platform (Figure 1). To allow phage entry into mammalian cells to achieve gene delivery, we evaluated the bacteriophage vector system in combination with chemical modifications of the phage capsid using transduction-enhancing agents. Three different types of transduction reagents were tested including cationic lipids, polycationic polymers and calcium phosphate (Figure 1). We established optimal transduction conditions for the phage-reagent vector system and investigated the mechanisms by which increased gene delivery efficacy was achieved. Integration of phage with traditional transfection reagents resulted in increased ability of phage binding to the cell surfaces, entry into cells and subsequent efficient gene expression by the phage vectors. Moreover, we determined that gene delivery by the hybrid vector, consisting of cationic polymers, was highly efficient in stable cell line generation. Finally, importantly, we demonstrated that integration of both chemical modification and genetic engineering of the phage capsid through display of the RGD4C ligand resulted in further increase of phage-mediated gene delivery compared to each modification alone. Here, we propose that these hybrid phage complexes have the potential to be an industry wide tool for high gene delivery output as well as applications within laboratory research.

## 2. Materials and Methods

### 2.1. Construction and Production of Bacteriophage Vectors

As phage vectors, we used the previously reported AAVP vector. To construct these vectors, phage was genetically manipulated to carry a mammalian transgene cassette encoding the cytomegalovirus (CMV) promoter-driven transgene, flanked by inverted terminal repeats (ITR)s from AAV2. Some phage vectors were also further manipulated for ligand-mediated gene delivery by incorporating copies of the RGD4C tumor targeting peptide on the pIII minor coat protein of the phage, in order to generate the RGD4C-phage. Phage vectors, with RGD4C (RGD4C-phage) or without RGD4C (phage), were amplified, isolated and purified from the culture supernatant of host bacteria (*Escherichia coli* K91) as previously described [13]. Phage particles were sterile filtered through 0.45 µm filters, then titrated using a qNano analyzer (IZON Science Ltd. T., Oxford, UK) based on a coulter technique also known as resistive pulse sensing, and expressed as mg/mL.

### 2.2. Chemical Modification of Phage Vectors

Three different types of transfection reagents were tested in combination with the phage vectors including cationic lipids, cationic polymers and calcium phosphate (CaPi). For cationic lipids and polymers, desired concentrations of chemicals were added to 25 μg of phage vector preparations in complete Dulbecco’s Modified eagle’s Medium (DMEM, Sigma, Dorset, UK). Solutions were incubated for 15 min at room temperature to allow for the formation of complexes before initiation of cell transduction. For calcium phosphate, the desired concentration of CaCl_2_ was added to 25 μg phage vector prepared in double distilled water. An equal volume of 2× Hepes-buffered saline solution (HBS) was added to allow the formation of precipitates.

### 2.3. Cell Culture 

Human Embryonic Kidney (HEK293) cell line was purchased from American Type Culture Collection (ATCC, Manassas, VA, USA). The human MCF-7 breast cancer cell line was from the Cancer Research UK (London, UK)). Cells were cultured in DMEM, supplemented with 10% Fetal Bovine Serum (FBS, Sigma), Penicillin (100 units/mL, Sigma), Streptomycin (100 μg/mL, Sigma) and l-Glutamine (2 mmol/mL, Sigma). Cells were maintained in a humidified atmosphere of 37 °C and 5% CO_2_ and passaged every 3–4 days once they reach 70%–80% confluence.

### 2.4. In Vitro Cell Transduction by Phage-Derived Vector Complexes

Cells were seeded at 3 × 10^4^ cells/well in 48-well plates and allowed to proliferate until 60%–80% confluent. Complexes of phage vectors and transfection reagents prepared at optimal ratios in serum-free media, or control phage vector alone were applied to the cells, followed by incubation at 37 °C for 4 h. Next complete media, containing FBS, was administered and the cells were incubated at 37 °C to allow for transgene expression. Transduction efficiency of the phage complexes was determined by using phage carrying the green fluorescent protein (*GFP*) or firefly luciferase (*Luc*) reporter genes. GFP expression was evaluated using a Nikon eclipse TE200-S fluorescent microscope (Nikon, Surrey, UK). *Luc* reporter gene expression in transduced cells was determined by using the Promega Steady-Glo^®^ Luciferase Assay kit following the manufacturer’s protocol and quantified using a Promega plate reader (Promega, Southampton, UK). Data were normalized to 100 µg protein levels as determined by the Bradford assay and presented as relative luminescence units per 100 µg protein. Cell viability was analyzed by CellTiter-glo^®^ cell viability assay kit; following the manufacturer’s protocol and quantified using a Promega plate reader. All cell transduction experiments were repeated three times and performed in triplicates.

### 2.5. In Vitro Depletion Assay

HEK293 cells in 48-well plates, at 70%–80% confluence, were treated with phage vectors prepared at optimal ratios with the desired transfection reagents. The plates were placed on ice for 1 h to prevent internalization followed by collection of supernatants, which were then subjected to serial dilution in 1× phosphate buffered saline (PBS) solution. Experiments were performed in triplicates, repeated twice, and K91 Kan bacterial infection method was used to quantify the number of phage vector particles by counting transducing units as previously reported [13].

### 2.6. Internalization Assay

Phage vector particles internalized in HEK293 cells were quantified as previously reported [20]. Briefly, unbound and surface bound vector particles were removed from cells by washing with 1× PBS and trypsin, respectively. Cells were centrifuged at 200 rpm for 5 min and fixed in 4% paraformaldehyde (PFA) for 10 min at room temperature. Cells were blocked in 0.1% saponin in 2% bovine albumin serum (BSA)-PBS for 30 min. To detect internalized phage, cells were stained with a rabbit anti-M13 phage antibody (dilution 1:1000) in 0.1% saponin in 1% BSA-PBS for 1 h at room temperature. Cells were centrifuged and resuspended in 0.1% saponin in 1% BSA-PBS followed by incubation with a goat anti-rabbit AlexaFluor-647 (dilution 1:500) for 1 h at room temperature. For analysis, cells were washed in 0.1% saponin-PBS and resuspended in PBS. Fluorescence-activated cell sorting analysis was carried out using a FACSCalibur Flow cytometer (BD Biosciences, Oxford, UK) equipped with an argon-ion laser (488 nm) and red-diode laser (365 nm). Experiments were repeated twice, and 10,000 gated cells per triplicate wells were used as the mean fluorescence intensity. Results were analyzed using Flowjo software (TreeStar Ashland, OR, USA).

### 2.7. Generation of Stable Cell Lines

HEK293 and MCF-7 monolayer cell cultures in 12-well plates were treated with phage vectors carrying *GFP* and a puromycin-resistant gene (*puro*^r^), combined or not with transfection reagents. At day 3 post-transduction, cells were trypsinised and resuspended in DMEM containing 1 μg/mL puromycin antibiotic, which was replenished every 3 days. After two weeks, puromycin-resistant clones were visible via fluorescent microscope and were subsequently pooled and passaged to generate a population of stably transduced cells.

### 2.8. Confocal Microscopy

Cells were seeded on 18 mm^2^ coverslips in 12-well plates. Cells were incubated with phage vectors (prepared at optimal ratios with transfection reagents) for 4 h, washed with PBS and fixed in 4% PFA in PBS for 15 min at room temperature and quenched with 50 mmol/L ammonium chloride. Cells were permeabilized with 0.2% Triton X-100, washed and blocked with 2% BSA-PBS for 30 min. For phage staining, cells were incubated for 1 h with rabbit anti-M13 phage (1:1000) followed by secondary AlexaFluor-conjugated antibodies (dilution 1:750) with/without DAPI (dilution 1:2000) for 1 h at room temperature. Finally, cells were mounted in Mowiol mounting medium and images were acquired with a Leica SP5 confocal microscope (Leica, Milton Keynes, UK). Experiments were performed in triplicates and repeated twice.

### 2.9. Statistical Analysis

GraphPad Prism software (version 5.0, GraphPad Software, Inc., La Jolla CA, USA) was used to perform statistical analyses Error bars represent standard error of the mean (s.e.m). *p* values were generated by ANOVA (Analysis of Variance) and denoted as follows: *p < 0.05, **p <0.01, ***p < 0.001 and n.s., non-significant.

## 3. Results

### 3.1. Cationic Transfection Reagents Mediate Significant Gene Transfer by Bacteriophage in HEK293 Cells

**Figure 2:**
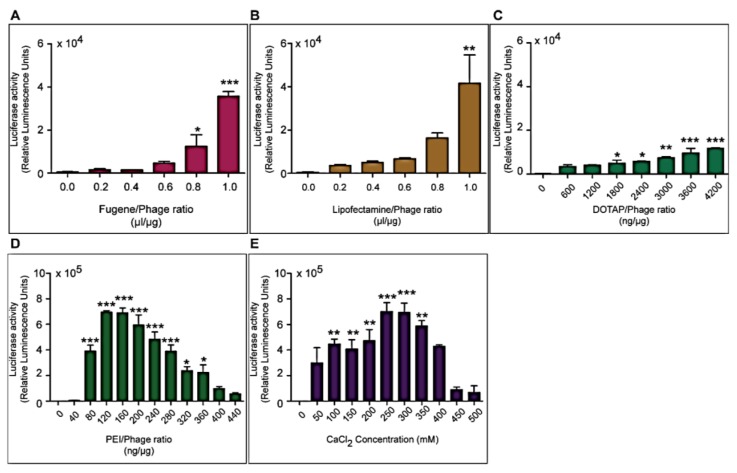
Optimization of transfection reagents for enhanced phage-mediated gene transfer in HEK293 cells. HEK293 cells were treated with phage, carrying the *Luc* reporter gene, premixed with increasing concentrations of lipid-based reagents (**A**. Fugene 6; **B**. Lipofectamine or **C**. DOTAP); the cationic polymer PEI (**D**) or calcium phosphate (**E**). Luciferase activity was analyzed at day 3 post-transduction. Results represent the average relative luminescence units of triplicate wells and error bars represent the standard error of the mean. Asterisks are defined as: * *p* < 0.05, ** *p* <0.01, *** *p* < 0.001.

We sought to assess whether the integration of phage-derived vectors with conventional transfection reagents can mediate and improve the phage gene transfer efficiency to eukaryotic cells. Therefore, we examined the efficacy at which phage/transfection reagent complexes transduce the human embryonic kidney HEK293 cells, as these cells are extensively used for general DNA transfection and viral transduction purposes and have also previously been used as a standard *in vitro* model for phage-mediated gene delivery [13,14]. Three different types of transfection reagents were tested: cationic lipids, cationic polymers and calcium phosphate. The efficiency of transfection depends largely on the vector to chemical ratio and so we first needed to determine the optimal ratios for our novel hybrid vector system [21]. Cells were transduced with phage vector carrying the firely luciferase *(Luc*) reporter gene, with or without transfection reagents at different phage/transfection reagent ratios. Analysis of *Luc* expression at 72 h post-transduction revealed that *Luc* expression dramatically improved with increased concentrations of reagents compared to phage alone (no reagent, Figure 2). For the lipid-based reagents, maximum levels of transgene expression were achieved at lipid/phage ratios of 1 µL/µg for Fugene 6 and Lipofectamine, and 4200 ng/µg for DOTAP (*N*-[1-(2,3-Dioleoyloxy)propyl]-*N*,*N*,*N*-trimethyl-ammonium methyl-sulfate) with overall increases of 70-, 80- and 20-fold, respectively (Figure 2A, B and C). For the cationic polymer polyethylenimine (PEI) and calcium phosphate (CaPi) (Figure 2D and E, respectively), *Luc* expression increased with increasing concentrations of reagents reaching a maximum at 160 ng/µg for PEI and 250 mM for CaCl_2_, followed by a gradual decrease.

Cell viability assays were performed to determine whether the decrease in luciferase activity was caused by cytotoxicity due to high amounts of the transfection reagents. Minimal cytotoxicity was observed for all transfection reagents apart from PEI where higher concentrations above 280 ng/µg were toxic and subsequently contributing to the reduction in *Luc* expression (data not shown). The reagents PEI and calcium phosphate elicited higher transduction efficiencies compared to the lipid based reagents reaching values of ~7 × 10^5^ compared to 4 × 10^4^ relative luminescence units, respectively. Therefore, PEI and calcium phosphate were selected for further studies of phage-mediated gene delivery.

**Figure 3:**
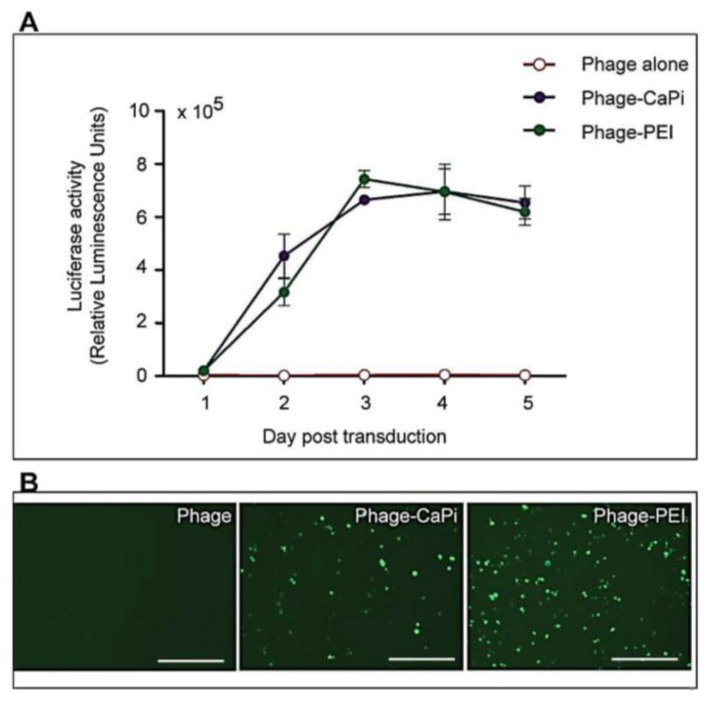
Kinetic study of phage combined with calcium phosphate and PEI in HEK293 cells. HEK293 cells were treated with phage vectors carrying the *Luc* or *GFP* reporter genes, premixed with optimized ratios of calcium phosphate or PEI and compared to phage vector alone. (**A**) The luciferase assay was performed daily over a time period of five days following vector transduction. Results represent the average relative luminescence units of triplicate wells and error bars represent the standard error of the mean; (**B**) Representative images showing *GFP* expression mediated by phage-CaPi and phage-PEI in HEK293 cells, taken at day 5 post-transduction. Scale bars = 500 µm.

Next we used optimized ratios of PEI or calcium phosphate and phage carrying the *Luc* reporter gene to assess the efficacy of gene transfer by the hybrid phage/transfection reagent vector system over a period of five days following transduction of HEK293 cells (Figure 3A). Phage vector alone was used as a control. A significant increase in *Luc* gene expression was detected in cells transduced with hybrid phage-PEI and phage-CaPi, which increased rapidly over the time course compared to phage vector alone, which didn’t elicit any detectable levels of *Luc* gene expression. For instance, at day 5 post-transduction, treatment with phage-CaPi or phage-PEI resulted in ~7- or ~6 -fold increase, respectively, in *Luc* expression compared to phage vector alone. Fluorescent microscopy images of HEK293 cells treated with phage vectors carrying the green fluorescent protein *(GFP*) reporter gene further highlight the increase in reporter gene expression elicited by the phage-CaPi and phage-PEI complexes, compared to phage alone, where no GFP expression was detected (Figure 3B).

These data suggest that the integration of conventional transfection reagents with phage vectors boosts gene transfer efficacy. Furthermore, not all transfection reagents increase phage-mediated gene transfer to the same extent. It is clear that combination with calcium phosphate and PEI resulted in the highest increase compared to the lipid-based reagents.

### 3.2. Mechanisms of Phage-Mediated Gene Transfer by CaPi and PEI

**Figure 4:**
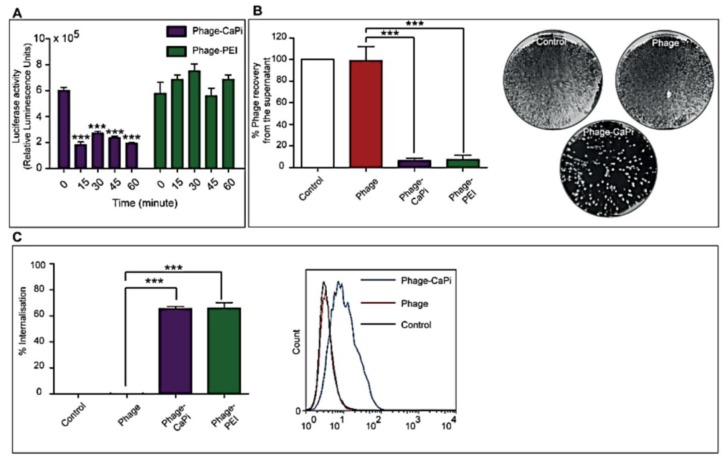
Investigation of cell transduction mechanisms mediated by phage-CaPi and phage-PEI complexes. HEK293 cells were treated with phage premixed with optimized concentrations of CaPi and PEI. (**A**) Effect of complex formation time before addition to cells. Phage-CaPi or phage-PEI was incubated for various times before being applied to cells. Experiments were performed in triplicate; (**B**) Depletion assay analysis of free cell-unbound phage particles from the supernatant of transduced cells. Photographs representing colony count of *E. coli* cells from the depletion assay; (**C**) Evaluation of internalization efficiency of hybrid phage complexes. Flow cytometric analysis of uptake of phage-CaPi and phage-PEI complexes or control phage alone. Asterisks are defined as: *** *p* < 0.001.

To gain insight into the mechanisms responsible for the observed increase in gene delivery efficacy by CaPi and PEI, we sought ways to explore the interactions between the phage/reagent complexes and the cell surface. The data in Figure 4A show the duration of complex formation between phage and CaPi or PEI. Cell treatment with phage-CaPi, immediately after preparation, elicited an optimal effect on transduction of HEK293 cells compared to phage-PEI, which efficacy remained intact over a 60 min time course post preparation that we have tested in this experiment.

As we previously reported, an important rate-limiting factor of phage-mediated gene transfer is the lack of virus binding to the cell surface [22]. Therefore, we tested the hypothesis that the enhanced efficacy of phage vectors, combined with transfection reagents, is likely due to effects on virus adsorption to target cell membranes. We performed a supernatant depletion assay to determine the amount of free cell-unbound phage, which remains in the external fluid phase above the adherent cell layer by infection of host bacteria followed by colony counting (Figure 4B). Less than 10% input phage particles were recovered from HEK293 cells incubated with phage-CaPi or phage-PEI, indicating that ~90% of phage was bound to the surface of cells. In contrast, almost 100% of input phage was recovered from cells treated with phage alone. No phage depletion was observed in the supernatant of control cells that didn’t receive any phage treatment. Next, internalization assays by flow cytometry revealed that CaPi and PEI significantly increased cellular entry of phage vectors into HEK293 by approximately 60% compared to entry of the phage vectors in the absence of CaPi or PEI (Figure 4C).

**Figure 5:**
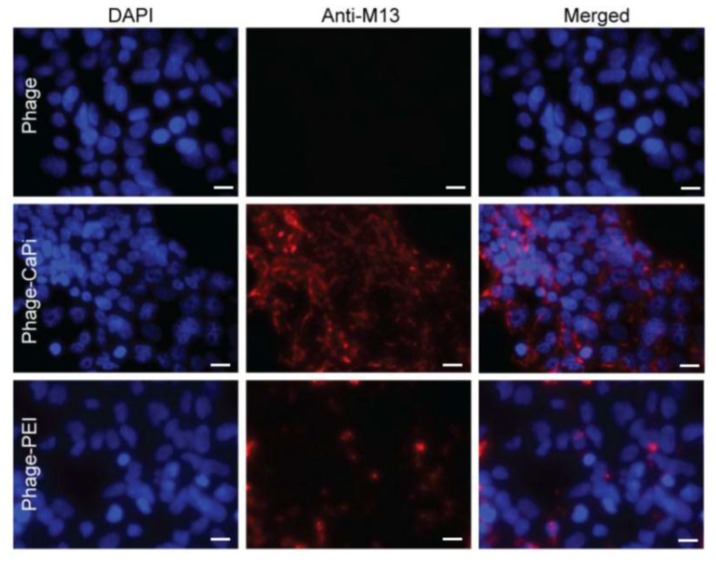
Phage-CaPi and phage-PEI complexes showed enhanced cell surface localization. HEK293 cells were treated with optimized ratios of phage premixed with either CaPi or PEI. Co-localization of complexes with cells was analyzed by using confocal microscopic imaging. Phage vector particles were double immunofluorescence stained with a rabbit anti-M13 phage primary antibody followed by staining with goat anti-rabbit AlexaFluor-594 secondary antibody (red). Nuclei were stained with DAPI (blue). Scale bar = 10 µm.

Confocal microscopic imaging of HEK293 cells following immunofluorescence with an anti-phage antibody further highlights the greater cell surface localization of the phage-CaPi and phage-PEI complexes (phage appears in red) compared to phage alone, where no phage particles were observed on cells (Figure 5). Altogether, these data suggest that inclusion of phage in a complex with CaPi or PEI mediates gene transfer to mammalian cells, at least in part, by allowing both phage binding to the cell surface and its subsequent cellular entry.

### 3.3. Ligand-Directed Phage Transduction Further Enhances Gene Transfer by the Hybrid Phage/Transfection Reagent System

**Figure 6:**
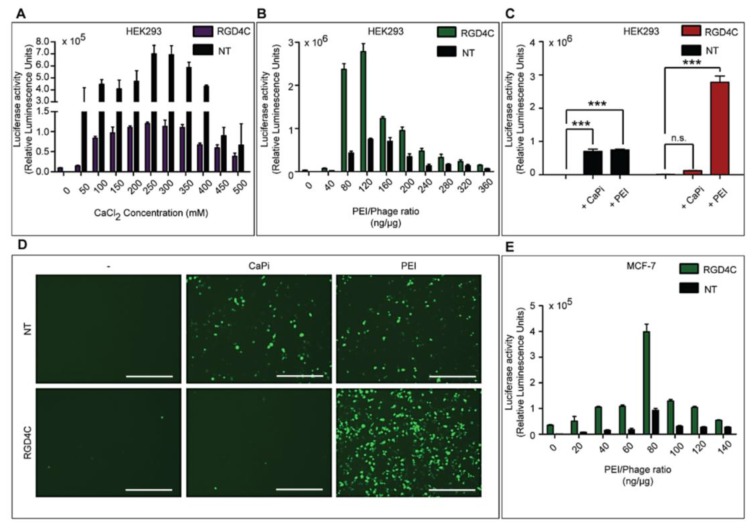
Combination of chemical and genetic modification boosts phage-mediated gene transfer. HEK293 cells were treated with targeted RGD4C-phage vectors or non-targeted-phage vectors, carrying the *Luc* or *GFP* reporter genes, premixed with CaPi or PEI. (**A**) Optimization of CaCl_2_ concentrations with RGD4C-phage vectors in HEK293 cells; (**B**) Optimization of PEI concentrations with RGD4C-phage vectors in HEK293 cells; (**C**) Comparison of transduction efficiency between RGD4C-phage and non-targeted (NT) phage vectors premixed with either CaPi or PEI; (**D**) Fluorescent imaging of HEK293 cells treated with vectors carrying the *GFP* reporter gene to show comparison between non-targeted, NT, and targeted phage, RGD4C, complexed with either CaPi or PEI. Scale bars = 500 µm; (**E**) Optimization of PEI concentrations with RGD4C and NT phage vectors in MCF-7 cells. Asterisks are defined as: *** *p* < 0.001 and n.s., non-significant.

Having shown that chemical modification of phage vectors with either CaPi or PEI increases gene delivery efficiency; we next sought to determine whether incorporation of genetic targeting into the vector complexes would lead to further enhancement of gene transfer. Our previously reported phage vectors, AAVP, were initially designed to enter mammalian cells and allow gene expression by ligand display on the phage capsid to bind to specific mammalian receptors [23,24]. The targeting peptide ligand, cyclic RGD4C, was engineered to be displayed on the pIII minor coat protein of phage to allow the vector (RGD4C-phage) to enter mammalian cells expressing αv integrin receptors [13]. We therefore investigated the effect of integration of increasing concentrations of CaCl_2_ or PEI with targeted RGD4C-phage vectors, carrying the *Luc* reporter gene, in HEK293 cells (Figure 6A,B, respectively). HEK293 cells were chosen as they display αv integrins and have previously been used for investigation of targeted RGD4C-phage vectors [13,14]. Analysis of *Luc* reporter gene expression, at day 3 post-transduction, revealed that transduction efficiency of RGD4C-phage was significantly increased when combined with either CaPi and PEI as compared to combinations of CaPi and PEI with non-targeted phage (NT, lacking RGD4C). Maximum transduction efficiencies were obtained at 250–300 mM CaCl_2_ and 120ng/µg PEI per RGD4C-phage ratio, followed by a gradual decrease in *Luc* gene expression.

The relative transduction efficiencies of numerous vector systems were assessed: non-targeted phage (NT), targeted RGD4C-phage (RGD4C) and both phage types combined with either CaPi or PEI, using the optimized ratios of each transfection reagent (Figure 6C). Complexes of non-targeted phage and CaPi at an optimal ratio dramatically increased *Luc* reporter gene expression up to 1300‑fold compared to non-targeted phage alone. Transgene expression was also significantly increased to 1500-fold when we combined PEI with non-targeted phage at an optimal ratio of 160 ng/μg (Figure 6C). Incorporation of targeted RGD4C-phage with an optimal concentration of PEI greatly increased *Luc* reporter gene expression, with a maximal 5500-fold increase compared to the control non-targeted phage (Figure 6C). Figure 6D provides representative data of the hybrid complexes, carrying the *GFP* reporter gene and further highlights the superiority of the hybrid phage vector systems.

These data demonstrate that PEI is the superior transfection reagent for enhancing phage gene transfer efficacy in combination with ligand-mediated phage transduction compared to CaPi as it increases transduction efficiency of both non-targeted and targeted RGD4C-phage vectors. Although cell surface localization of the phage-CaPi was greater than phage-PEI (Figure 5), internalization experiments did not show any significant difference in cell entry (Figure 4C). Therefore, the phage-PEI might bestow advantage in gene expression by improving the intracellular trafficking of the phage, such as endosomal escape.

To demonstrate that our hybrid vector system is not cell line specific we used the RGD4C-Phage-PEI vector complex to determine its transduction efficiency in the MCF-7 human breast cancer cell line, as these cells have previously been used as a model for RGD4C-phage gene transfer [25]. Optimization of PEI complexes revealed that transduction efficiency was greatest with a PEI/phage ratio of 80 ng/μg (Figure 6E).

### 3.4. Stable Cell Lines Generation by the Hybrid Phage/Transfection Reagent Complex

**Figure 7:**
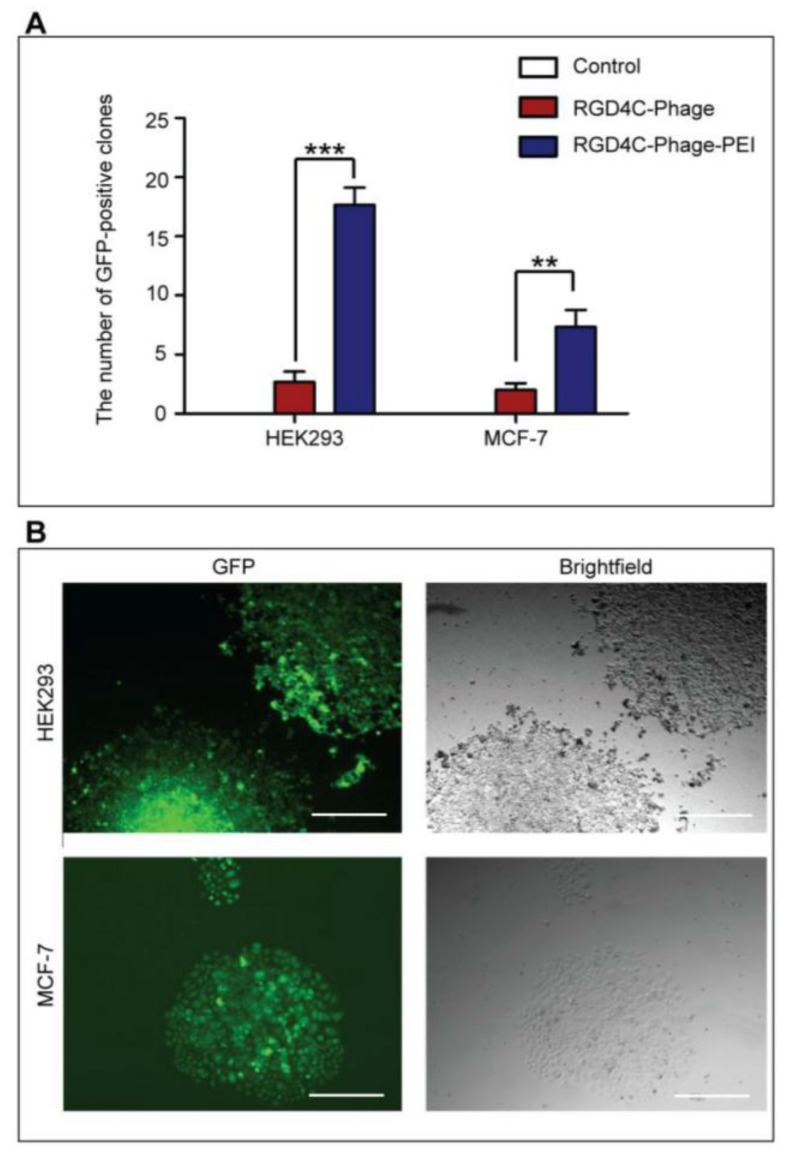
Generation of stable cell lines using RGD4C-Phage-PEI complexes. HEK293 and MCF-7 cells were treated with RGD4C-Phage vectors, carrying the *puro*^r^ gene and the *GFP* reporter gene, premixed with optimized ratios of PEI and compared to RGD4C-phage alone. Stably transduced cells were selected by puromycin resistance for 14 days post-transduction. Clones were pooled and passaged to generate stable cell lines. (**A**) Yield of puromycin-resistant colonies in HEK293 and MCF-7 cells; (**B**) Representative images of puromycin-resistant and GFP-expressing clones after 20 passages. Scale bars = 500 µm. Asterisks are defined as: ** *p* <0.01 and *** *p* < 0.001.

After establishing that integration of the PEI transfection reagent with the ligand-directed RGD4C-phage vector provides substantial gene transfer efficiency, we attempted to utilize our hybrid vector system for a specific research application by determining whether transgene expression could be sustained for the generation of stable cell lines. To test the efficiency of transgene expression of the RGD4C-Phage-PEI complex, we constructed RGD4C-phage vector carrying the puromycin-resistant gene (*puro*^r^) in addition to the *GFP* reporter gene. When this vector is transfected into cells and incubated in media containing the antibiotic puromycin, only cells expressing the resistant transgene survive. As proof-of-concept, we compared the RGD4C-Phage-PEI complex with RGD4C-phage alone in both HEK293 and MCF-7 cells in order to rule out the possibility that our vector system was particular to one cell line. Transduced cells were grown in media supplemented with 1 μg/mL puromycin and stably transduced cells were monitored using a fluorescent microscope. Non-transduced cells were used as controls, which were all killed within a few days. After 14 days, the puromycin-resistant cell clones were counted with and without a GFP filter on the microscope. The RGD4C-Phage-PEI complexes resulted in a significantly higher number of puromycin-resistant clones in both HEK293 and MCF-7 cells compared to cells transduced with target RGD4C-phage alone (Figure 7A). For example, in HEK293 cells the number of resistant-clones was 18 ± 1 clones/well for RGD4C-Phage-PEI compared to 3 ± 1 clones/well for RGD4C-Phage alone.

Resistant clones were isolated, pooled and incubated in media without antibiotics then passaged for at least 20 passages in order to investigate the stability of *GFP* reporter gene expression. *GFP* expression was maintained through all passages of HEK293 and MCF-7 cells (Figure 7B). Therefore, these data indicate the effectiveness of the hybrid RGD4C-Phage-PEI complex as a suitable vector system for the generation of stable cell lines and show promise for other gene transfer applications.

## 4. Discussion

The delivery of nucleic acids into mammalian cells through non-viral methods has origins as far back as the 1960s [26]. The ultimate goal of transfection is to deliver nucleic acids into cells so as to investigate gene function. This goal can be accomplished by expression of exogenous genes. Manipulation of gene expression is a core technique in research areas such as drug development, cancer research, gene therapy, and tissue engineering [27,28]. Additionally, *in vivo* gene therapy applications have created a need to develop safer and more efficient techniques for delivering nucleic acids to different organs and tissues [8]. This *in vivo* research requires proof of concept, which is made possible through *in vitro* transfection experiments.

Calcium phosphate represents the oldest and most inexpensive chemical method for transfecting nucleic acids [4]. Despite the simple and cost-effective nature of the calcium phosphate method, it is ineffective for hard-to-transfect cells, is very sensitive to changes in pH, often lacks reproducibility, and requires large quantities of DNA. Other gene delivery methods that were developed after the introduction of calcium phosphate methods include direct delivery via injection into the cell nucleus (microinjection), use of viral vectors, electrical currents and lipid mediated techniques. Although microinjection provides a direct method for delivery of nucleic acid for cells that are difficult to transfect, it is low-throughput and a technique that is difficult to master. Electroporation has also been introduced and relies on an electrical field to transiently increase cell permeability; however, the application of an electrical field causes substantial cytotoxicity.

An alternative and more efficient method for delivering nucleic acid into cells is the use of viral vectors [3]. The use of viral vectors is a common methodology for efficiently delivering nucleic acid, especially in hard-to-transfect cells. Generation of recombinant viruses requires the packaging of exogenous DNA within the viral genome and subsequent delivery through infection of the target cell. The use of viral vectors was used as early as the late 1970s to express functional mRNA and protein. Although viral transduction is an efficient and effective option, some disadvantages include viral recombination, off-target effects, immune response induction, and possible oncogenic effects [29].

In brief, gene delivery continues to play a major role in a wide range of applications; however, the disadvantages of current gene transfer strategies necessitate more robust methods for nucleic acid delivery. Bacteriophage which has been recently described as a promising new generation of gene delivery vectors to mammalian has faced limited progress. Indeed, phage has no tropism for mammalian cells and thus cannot enter these cells to deliver gene expression. Genetic engineering of the phage has been shown to mediate gene delivery by display of ligands on the phage capsid to allow binding to a mammalian receptor and subsequent internalization of phage in mammalian cells. Herein, we show that chemical modification of the phage capsid allows phage entry into mammalian cells and subsequent expression of the gene of interest. Importantly, combination of both genetic and chemical modifications of the phage capsid further enhances gene delivery compared to each individual approach. Actually, we have developed a simple and effective method to enhance transduction efficiency of bacteriophage-based vectors using a combination of both conventional transfection reagents and ligand-directed transduction. Chemical modification has been combined with eukaryotic viral vectors to transfer genes to mammalian cells [30,31]. Genetic modification has also been applied to animal viral vectors to improve specificity; but to our knowledge, this is the first report showing combination of these two modifications into one single gene delivery particle. Moreover, there have been no reports regarding the application of filamentous bacteriophage complex with CaPi for gene transfer. The system we have described here has a number of advantages, and the results are encouraging.

In conclusion, we have presented a novel strategy to advance phage-based gene transfer by combining phage with conventional transfection reagents. Importantly, we report that integration of chemical modification with ligand-directed phage transduction further enhances phage-mediated gene delivery. This proof-of-concept study successfully shows that bacteriophage-mediated gene transfer into mammalian cells can be improved. Future studies to assess efficacy of these phage complexes in additional cell lines and applications, should provide further characterization of these hybrid phage complexes.
